# 1-Bromo-4-chloro-2,5-dimethoxy­benzene

**DOI:** 10.1107/S1600536810000504

**Published:** 2010-01-13

**Authors:** Yang Song, Sean Parkin, Hans-Joachim Lehmler

**Affiliations:** a College of Pharmaceutical Sciences, Southwest University, Chong Qing 400716, People’s Republic of China; b Department of Chemistry, University of Kentucky, Lexington, KY 40506-0055, USA; c Department of Occupational and Environmental Health, The University of Iowa, 100 Oakdale Campus, 124 IREH, Iowa City, IA 52242-5000, USA

## Abstract

The mol­ecule of the title compound, C_8_H_8_BrClO_2_, sits on a crystallographic inversion centre, which ensures that the halogen sites are disordered, with exactly 50% Br and 50% Cl at each halogen site. The inversion renders the two meth­oxy groups equivalent. These groups lie almost in the plane of the aromatic ring system, making dihedral angles of 8.8 (4)° to the ring.

## Related literature

For the synthesis of PCBs and PCB metabolites using the Suzuki coupling reaction, see: Lehmler & Robertson (2001 ▶); Song *et al.* (2008 ▶). For similar structures of halogenated meth­oxy-benzenes, see: Rissanen *et al.* (1988 ▶); Telu *et al.* (2008 ▶) and literature cited therein. For general background about PCBs, see: Hansen (1999 ▶); Robertson & Hansen (2001 ▶).
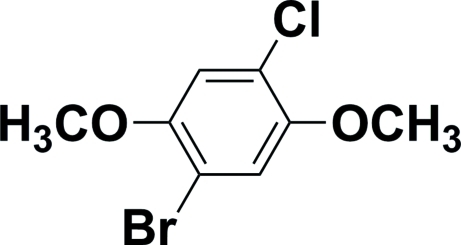


## Experimental

### 

#### Crystal data

C_8_H_8_BrClO_2_
*M* *_r_* = 251.50
Monoclinic, 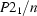

*a* = 6.3804 (7) Å
*b* = 8.2586 (10) Å
*c* = 8.6337 (11) Å
β = 90.853 (6)°
*V* = 454.89 (9) Å^3^
*Z* = 2
Mo *K*α radiation
μ = 4.77 mm^−1^
*T* = 90 K
0.25 × 0.22 × 0.22 mm

#### Data collection

Nonius KappaCCD diffractometer
Absorption correction: multi-scan (*SADABS*; Sheldrick, 1996 ▶) *T*_min_ = 0.310, *T*_max_ = 0.350
1938 measured reflections
1021 independent reflections
824 reflections with *I* > 2σ(*I*)
*R*_int_ = 0.032

#### Refinement

*R*[*F* ^2^ > 2σ(*F* ^2^)] = 0.030
*wR*(*F* ^2^) = 0.069
*S* = 1.04
1021 reflections
61 parameters
2 restraints
H-atom parameters constrained
Δρ_max_ = 0.57 e Å^−3^
Δρ_min_ = −0.49 e Å^−3^

### 

Data collection: *COLLECT* (Nonius, 1998 ▶); cell refinement: *SCALEPACK* (Otwinowski & Minor, 1997 ▶); data reduction: *DENZO-SMN* (Otwinowski & Minor, 1997 ▶); program(s) used to solve structure: *SHELXS97* (Sheldrick, 2008 ▶); program(s) used to refine structure: *SHELXL97* (Sheldrick, 2008 ▶); molecular graphics: *XP* in *SHELXTL* (Sheldrick, 2008 ▶); software used to prepare material for publication: *SHELX97* and local procedures.

## Supplementary Material

Crystal structure: contains datablocks I, global. DOI: 10.1107/S1600536810000504/si2231sup1.cif

Structure factors: contains datablocks I. DOI: 10.1107/S1600536810000504/si2231Isup2.hkl

Additional supplementary materials:  crystallographic information; 3D view; checkCIF report

## Figures and Tables

**Fig. 1.**
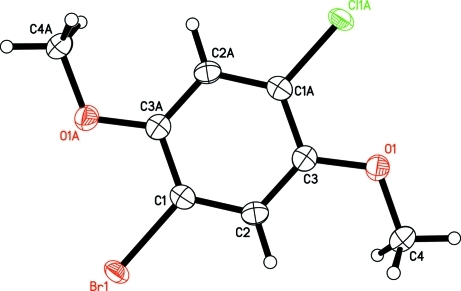
View of the title compound showing the atom-labelling scheme. Displacement ellipsoids are drawn at the 50% probability level. Atoms labelled A are inversion related, symmetry code A: 1 - x, 1 - y, 1 - z. The disordered atoms ClB and BrB have been omitted for clarity.

## supplementary crystallographic information

### Comment

The title compound is a precursor for the synthesis of hydroxylated metabolites of polychlorinated biphenyls (PCBs) (Lehmler & Robertson, 2001; Song *et al.*, 2008), a class of abundant, persistent organic pollutants in the global ecosystem (Hansen, 1999; Robertson & Hansen, 2001). The molecule of the title compound sits on a crystallographic inversion centre, which ensures that the Br/Cl sites are disordered exactly 50:50 in very nearly the same position. Furthermore, the inversion renders the two methoxy groups equivalent. The chlorine, bromine and the two methoxy groups essentially lie within the plane of the benzene ring, with the dihedral angles between the plane of the benzene ring (C1 through C3 and C1A through C3A) and the methoxy group being 8.8 (4)° (for both methoxy groups). This is comparable to the solid state conformation of the methoxy group of other methoxybenzenes with none or only one *ortho* substituent (Rissanen *et al.*, 1988). Overall, these structural characteristics make the title compound a perfect precurusor for the synthesis of PCB derivatives using the Suzuki coupling reaction (Song *et al.*, 2008).

### Experimental

The title compound was synthesized by chlorination of 1-bromo-2,5-dimethoxy-benzene with HCl/H_2_O_2_ as chlorination reagent as described previously (Song *et al.*, 2008). Crystals suitable for X-ray diffraction were grown by slow evaporation of a saturated solution of the title compound in CHCl_3_.

### Refinement

H atoms were found in difference Fourier maps and subsequently placed in idealized positions with constrained C—H distances of 0.98 Å (C_Me_H) and 0.95 Å (C_Ar_H) with *U*_iso_(H) values set to either 1.5*U*_eq_ or 1.2*U*_eq_ of the attached C atom respectively. For the disordered halogen sites, the distances were refined subject to a condition whereby the C—Cl distance was restrained (using *DFIX* in *SHELXL97*) to be 0.916 times the C—Br length (International Tables, vol C). The halogen displacement parameters were constrained to be equal (EADP in *SHELXL97*).

### Crystal data

**Table:**

C_8_H_8_BrClO_2_	*F*(000) = 248
*M**_r_* = 251.50	*D*_x_ = 1.836 Mg m^−^^3^
Monoclinic, *P*2_1_/*n*	Mo *K*α radiation, λ = 0.71073 Å
Hall symbol: -P 2yn	Cell parameters from 1051 reflections
*a* = 6.3804 (7) Å	θ = 1.0–27.5°
*b* = 8.2586 (10) Å	µ = 4.77 mm^−^^1^
*c* = 8.6337 (11) Å	*T* = 90 K
β = 90.853 (6)°	Block, colourless
*V* = 454.89 (9) Å^3^	0.25 × 0.22 × 0.22 mm
*Z* = 2	

### Data collection

**Table:**

Nonius KappaCCD diffractometer	1021 independent reflections
Radiation source: fine-focus sealed tube	824 reflections with *I* > 2σ(*I*)
graphite	*R*_int_ = 0.032
Detector resolution: 18 pixels mm^-1^	θ_max_ = 27.3°, θ_min_ = 3.4°
ω scans at fixed χ = 55°	*h* = −8→8
Absorption correction: multi-scan (*SADABS*; Sheldrick, 1996)	*k* = −10→10
*T*_min_ = 0.310, *T*_max_ = 0.350	*l* = −11→11
1938 measured reflections	

### Refinement

**Table:**

Refinement on *F*^2^	Secondary atom site location: difference Fourier map
Least-squares matrix: full	Hydrogen site location: inferred from neighbouring sites
*R*[*F*^2^ > 2σ(*F*^2^)] = 0.030	H-atom parameters constrained
*wR*(*F*^2^) = 0.069	*w* = 1/[σ^2^(*F*_o_^2^) + (0.0274*P*)^2^ + 0.2316*P*] where *P* = (*F*_o_^2^ + 2*F*_c_^2^)/3
*S* = 1.04	(Δ/σ)_max_ = 0.001
1021 reflections	Δρ_max_ = 0.57 e Å^−^^3^
61 parameters	Δρ_min_ = −0.49 e Å^−^^3^
2 restraints	Extinction correction: *SHELXL97* (Sheldrick, 2008), Fc^*^=kFc[1+0.001xFc^2^λ^3^/sin(2θ)]^-1/4^
Primary atom site location: structure-invariant direct methods	Extinction coefficient: 0.010 (2)

### Special details

**Table:**

Experimental. The crystals were of surprisingly poor quality, with diffraction spots around 3° wide. Nevertheless, because the cell is quite small there were no problems with reflection overlap.
Geometry. All e.s.d.'s (except the e.s.d. in the dihedral angle between two l.s. planes) are estimated using the full covariance matrix. The cell e.s.d.'s are taken into account individually in the estimation of e.s.d.'s in distances, angles and torsion angles; correlations between e.s.d.'s in cell parameters are only used when they are defined by crystal symmetry. An approximate (isotropic) treatment of cell e.s.d.'s is used for estimating e.s.d.'s involving l.s. planes.
Refinement. Refinement of *F*^2^ against ALL reflections. The weighted *R*-factor *wR* and goodness of fit *S* are based on *F*^2^, conventional *R*-factors *R* are based on *F*, with *F* set to zero for negative *F*^2^. The threshold expression of *F*^2^ > 2σ(*F*^2^) is used only for calculating *R*-factors(gt) *etc*. and is not relevant to the choice of reflections for refinement. *R*-factors based on *F*^2^ are statistically about twice as large as those based on *F*, and *R*- factors based on ALL data will be even larger.The structure straddles an inversion centre but the molecule itself does not possess inversion point symmetry, thus it is forced to be disordered in the crystal. This disorder places Cl and Br atoms at 50% occupancy in essentially the same position. For refinement, the C—Cl distance was restrained to be 0.916 times that of the C—Br distance. The ratio used was taken from the International Tables volume C. Further, anisotropic displacement parameters for the Br and Cl atoms were made equal.

### Fractional atomic coordinates and isotropic or equivalent isotropic displacement parameters (Å^2^)

**Table:**

	*x*	*y*	*z*	*U*_iso_*/*U*_eq_	Occ. (<1)
Br1	0.7756 (12)	0.2627 (9)	0.7207 (9)	0.0235 (3)	0.50
Cl1	0.760 (3)	0.271 (2)	0.711 (2)	0.0235 (3)	0.50
O1	0.6036 (3)	0.5566 (2)	0.19443 (19)	0.0247 (4)	
C1	0.6144 (4)	0.3987 (3)	0.5927 (3)	0.0212 (6)	
C2	0.6749 (4)	0.4217 (3)	0.4409 (3)	0.0213 (6)	
H2	0.7945	0.3673	0.4023	0.026*	
C3	0.5597 (4)	0.5244 (3)	0.3457 (3)	0.0208 (6)	
C4	0.7634 (4)	0.4604 (3)	0.1242 (3)	0.0281 (6)	
H4A	0.8990	0.4839	0.1740	0.042*	
H4B	0.7701	0.4861	0.0136	0.042*	
H4C	0.7302	0.3454	0.1371	0.042*	

### Atomic displacement parameters (Å^2^)

**Table:**

	*U*^11^	*U*^22^	*U*^33^	*U*^12^	*U*^13^	*U*^23^
Br1	0.0261 (12)	0.0218 (8)	0.0226 (10)	0.0081 (6)	−0.0047 (6)	0.0023 (5)
Cl1	0.0261 (12)	0.0218 (8)	0.0226 (10)	0.0081 (6)	−0.0047 (6)	0.0023 (5)
O1	0.0299 (11)	0.0221 (10)	0.0222 (10)	0.0029 (8)	0.0045 (8)	0.0016 (7)
C1	0.0221 (14)	0.0181 (13)	0.0232 (14)	−0.0004 (10)	−0.0038 (10)	−0.0013 (10)
C2	0.0214 (14)	0.0165 (13)	0.0259 (14)	0.0000 (10)	0.0005 (10)	−0.0043 (10)
C3	0.0230 (14)	0.0162 (12)	0.0232 (13)	−0.0037 (10)	0.0012 (10)	−0.0016 (10)
C4	0.0278 (16)	0.0314 (15)	0.0254 (15)	0.0043 (12)	0.0069 (12)	−0.0011 (12)

### Geometric parameters (Å, °)

**Table:**

Br1—C1	1.872 (4)	C2—C3	1.385 (3)
Cl1—C1	1.727 (9)	C2—H2	0.9500
O1—C3	1.366 (3)	C3—C1^i^	1.392 (3)
O1—C4	1.434 (3)	C4—H4A	0.9800
C1—C2	1.385 (3)	C4—H4B	0.9800
C1—C3^i^	1.392 (3)	C4—H4C	0.9800
			
C3—O1—C4	116.95 (19)	O1—C3—C2	124.9 (2)
C2—C1—C3^i^	122.3 (2)	O1—C3—C1^i^	116.9 (2)
C2—C1—Cl1	119.2 (8)	C2—C3—C1^i^	118.2 (2)
C3^i^—C1—Cl1	118.5 (8)	O1—C4—H4A	109.5
C2—C1—Br1	118.8 (3)	O1—C4—H4B	109.5
C3^i^—C1—Br1	118.9 (3)	H4A—C4—H4B	109.5
C3—C2—C1	119.5 (2)	O1—C4—H4C	109.5
C3—C2—H2	120.2	H4A—C4—H4C	109.5
C1—C2—H2	120.2	H4B—C4—H4C	109.5
			
C3^i^—C1—C2—C3	0.1 (4)	C4—O1—C3—C1^i^	−171.2 (2)
Cl1—C1—C2—C3	−179.7 (8)	C1—C2—C3—O1	−180.0 (2)
Br1—C1—C2—C3	−178.7 (3)	C1—C2—C3—C1^i^	−0.1 (4)
C4—O1—C3—C2	8.7 (4)		

Symmetry codes: (i) −*x*+1, −*y*+1, −*z*+1.
